# Development and validation of a nomogram based on LASSO-logistic regression for predicting carotid atherosclerosis in patients with hypertension

**DOI:** 10.3389/fcvm.2025.1581074

**Published:** 2025-11-04

**Authors:** Xin-fu Cao, Ya-li Qiu, Zhen-hua Gu, Chao Tang, Xiao-long Li, Dao-hai Chen

**Affiliations:** ^1^ Department of Cardiology, Changzhou Affiliated Hospital of Nanjing University of Chinese Medicine, Changzhou, Jiangsu, China; ^2^ Department of Respiratory Medicine, Changzhou Third People’s Hospital, Changzhou, Jiangsu, China; ^3^ Changzhou Medical Center, Nanjing Medical University, Changzhou, Jiangsu, China; ^4^ Department of Cardiology, The Second Affiliated Hospital of Soochow University, Suzhou, China

**Keywords:** hypertension, carotid atherosclerosis, logistic regression analysis, nomogram, LASSO regression

## Abstract

**Background and objectives:**

Carotid atherosclerosis (CAS) is increasingly prevalent among hypertensive patients. This study aims to develop a predictive nomogram for CAS in hypertensive population.

**Methods:**

A total of 930 patients with hypertension were hospitalized in the Department of Cardiology of the Affiliated Hospital of Changzhou, Nanjing University of Chinese Medicine (August 2018–August 2024) formed the development cohort, categorized into CAS (156 individuals) and non-CAS (774 individuals) groups. Additionally, 398 hypertensive patients from the Department of Cardiology of the Second Affiliated Hospital of Soochow University served as the validation cohort (ratio 7:3), with 72 CAS individuals and 326 non-CAS individuals. LASSO regression initially identified key risk factors, followed by logistic regression for further analysis. The nomogram, constructed using the “rms” package in R 4.2.6, underwent internal validation via the 1,000 iterations of Bootstrap resampling. Model performance was evaluated through ROC curves, calibration curves, and decision curve analysis.

**Results:**

Eight significant risk factors—Age, history of smoking (Smoke), history of diabetes mellitus (DM), course of hypertension (Course), physical activity (PA), body mass index (BMI), low-density lipoprotein (LDL), and uric acid (UA)—were identified (*P* < 0.05), among which DM was the most important influencing factor. The nomogram demonstrated strong predictive accuracy, with AUC values of 0.858 [95% CI (0.798, 0.918)] in the development cohort and 0.808 [95% CI (0.740, 0.876)] in the validation cohort. Calibration curves closely aligned with the ideal model, and decision curve analysis indicated optimal predictive performance within a probability threshold range of 0.050–0.960.

**Conclusions:**

This study presents a robust nomogram for assessing CAS risk in hypertensive patients, offering a valuable tool for clinical risk evaluation.

## Graphical Abstract

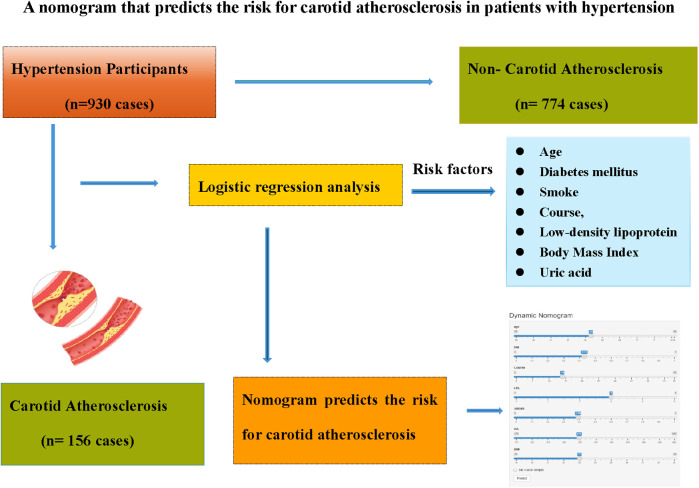

## Introduction

Hypertension is the most prevalent chronic cardiovascular disease globally, characterized by high incidence, low awareness, and poor control rates. Statistics indicate that 33% of individuals aged 30–79 suffer from hypertension (1), yet only 54% are diagnosed, 42% receive treatment, and merely 21% achieve effective control (2). As a major risk factor for ischemic heart disease, stroke, other cardiovascular conditions, chronic kidney disease, and dementia, hypertension imposes a substantial socioeconomic and public health burden (3, 4).

Carotid atherosclerosis (CAS), or carotid plaque, represents a localized manifestation of systemic atherosclerosis within the carotid artery. Early detection and standardized management of CAS are crucial for preventing ischemic stroke and systemic atherosclerosis. Research has established a strong link between CAS and hypertension. Globally, the incidence of both conditions is on the rise, and their coexistence is increasingly observed (5). Elevated blood pressure exerts excessive force on arterial walls, leading to endothelial cell retraction, structural disruption, and dysfunction. This process compromises vascular elasticity, resulting in arterial stiffening and thickening, ultimately contributing to CAS (6). Moreover, CAS exacerbates hypertension, while uncontrolled hypertension accelerates CAS progression, creating a self-perpetuating cycle (7, 8).

Studies reveal that hypertensive individuals with CAS face an increased risk of ischemic stroke (9). Identifying key CAS risk factors in this population and implementing preventive measures are essential for improving patient outcomes (10). While various studies have demonstrated that hypertension and CAS share common risk factors (11), there remains a scarcity of research focusing specifically on CAS risk factors among hypertensive patients. Previous research primarily focused on conventional risk factors such as diabetes mellitus, smoking, and hyperlipidemia, with less emphasis on clinical biomarkers like C-reactive protein (CRP), platelet count, and uric acid (UA). Additionally, no clinical prediction model currently exists for assessing CAS risk in hypertensive patients.

This study aims to bridge this gap by employing logistic regression analysis to identify CAS risk factors in hypertensive individuals and developing a predictive nomogram to serve as a clinical tool for risk assessment and prevention.

## Materials and methods

### Participants

A total of 930 hypertensive patients admitted to the Department of Cardiology at the Affiliated Hospital of Changzhou, Nanjing University of Chinese Medicine from August 2018 to August 2024 were included in the development cohort. In addition, 398 hypertensive patients from the Department of Cardiology of the Second Affiliated Hospital of Soochow University during the same period were collected in a ratio of 7:3 as the validation cohort for external validation of the model. Hypertension was diagnosed according to the Chinese Guidelines for the Prevention and Treatment of Hypertension (12). Participants were categorized into CAS and non-CAS groups based on the presence of CAS, with its diagnosis following the Guidelines for the Management of Atherosclerotic Carotid and Vertebral Artery Disease (13). The carotid intima-media thickness (IMT) of all patients was measured by a dedicated person using the GE LOGIQ9 color carotid ultrasound detector. The measurement method and standard were based on the Expert consensus on some problems of cerebral and carotid vascular ultrasonography (Part of carotid) (14). IMT < 1.0 mm was the normal IMT group, IMT ≥ 1.0 mm or IMT ≥ 1.2 mm at the bifurcation was the IMT thickening group, and IMT localization ≥1.5 mm, at least 0.5 mm greater than the surrounding normal IMT value, or greater than 50% of the surrounding normal IMT value was the IMT plaque group. CAS group = thickening group + plaque group.

### Inclusion criteria

(1) Those who met the diagnostic criteria of hypertension; (2) Those who underwent carotid color doppler ultrasound examination during hospitalization.

### Exclusion criteria

(1) Patients with incomplete medical records such as clinical tests and examinations; (2) Patients with poor compliance or lack of contact information.

### Study methods

A retrospective analysis was performed to gather clinical data from both patient cohorts, which included variables such as Age, Sex, history of smoking (Smoke), history of drinking (Drink), history of diabetes mellitus (DM, defined as physician-diagnosed diabetes, use of hypoglycemic agents, or HbA1c ≥ 6.5%), history of coronary heart disease (CHD, coronary CTA or coronary angiography shows at least one coronary artery stenosis greater than 50%), history of cerebral ischemic lesion (CIL, defined as an area of brain cell damage or necrosis caused by insufficient blood supply to local brain tissue, including lacunar cerebral infarction, focal cerebral infarction, and large-area cerebral infarction, confirmed by head CT and MRI), use of antihypertensive drugs (Antihypertensive), use of statins (Statins), use of antiplatelet agents (Antiplatelet), course of hypertension (Course, calculated from the earliest documented diagnosis), physical activity (PA, assessed via International Physical Activity Questionnaire short form), body mass index [BMI, calculated as weight(kg)/height(m)^2^ using calibrated scales and stadiometers], low-density lipoprotein (LDL), high-density lipoprotein (HDL), total cholesterol (TC), triglycerides (TG), CRP, platelet count (PLT), UA, and heart rate (HR), among others. Except for physical activity, which was completed through a questionnaire, all other clinical data were collected by reviewing the patients' electronic medical records.

Blood biomarkers (LDL, HDL, TC, TG, CRP, PLT, UA) were analyzed from fasting venous blood samples collected between 7:00 and 9:00 AM, processed within 2 h using Roche Cobas 8,000 analyzers. All biochemical assays followed manufactu rer protocols with internal quality controls.

Lifestyle data including “Drink” (defined as consuming at least one alcoholic beverage per month on average over the past 12 months) and “Smoke” (referring to smoking at least one cigarette per day for a cumulative period of six months or more) were extracted from standardized electronic health records supplemented by patient self-reported questionnaires administered during clinic visits. PA was quantified using the International Physical Activity Questionnaire short form, with metabolic equivalent-minutes/week categorized as low intensity (<600), Medium intensity (600–3,000), or high intensity (>3,000).

Data collection was independently carried out by two authors, with any discrepancies resolved by a third author. All procedures were conducted in accordance with the relevant guidelines and regulations.

### Statistical methods

The measurement data were analyzed using a Mann–Whitney *U* Test and presented as median (first quartile, third quartile), while the categorical variables were analyzed using *χ*^2^ or Fisher's exact test for expected counts <5. Continuous variables were standardized using Z-score normalization prior to LASSO regression. LASSO regression with 10-fold cross-validation was first employed to select key predictors from candidate variables (Age, Sex, DM, etc.). The optimal regularization parameter (lambda) was determined using lambda.1se = 0.15, minimizing both training and validation errors. Logistic regression analyses were carried out with SPSS 24.0. and subsequent analyses—including LASSO regression, ROC curve, calibration curve, and decision curve assessments—were executed using the “glmnet” and “rms” packages in R 4.2.6. Internal validation and consistency index calculations were performed through 1,000 iterations of Bootstrap resampling. A *p*-value < 0.05 was deemed statistically significant.

## Results

### Comparison of general data of patients in the development cohort and the validation cohort

A total of 930 hypertensive patients were enrolled in the development cohort, with 156 classified into the CAS group, resulting in an incidence rate of 16.75%. The validation cohort comprised 398 patients, of whom 72 had CAS, yielding an incidence rate of 18.21%. No statistically significant difference was observed between the incidence rates. There were significant differences in DM, Drink, TG, CRP, Antiplatelet and PLT between the two groups (*P* < 0.05), and there were no statistically significant differences in other baseline characteristics between the two groups (all *P* > 0.05), as shown in Table 1.

**Table 1:** Comparison of general information of patients in the development cohort and the validation cohort.

Variables	Development cohort (930 individuals)	Validation cohort (398 individuals)	Z/*χ*^2^	*P*-value
Age [years, M(Q1,Q3)]	57.00 (51.00,67.00)	59.00 (53.00, 66.00)	0.421	0.674
Male [*n* (%)]	496 (53.33)	208 (52.26)	3.230	0.072
DM [*n* (%)]	351 (37.74)	161 (40.45)	5.020	0.025
CIL [*n* (%)]	448 (48.17)	184 (46.23)	0.125	0.723
CHD [*n* (%)]	299 (32.15)	135 (33.91)	3.440	0.063
Drink [*n* (%)]	158 (16.98)	78 (19.59)	4.480	0.035
Smoke [*n* (%)]	202 (21.72)	92 (23.11)	3.834	0.052
Antihypertensive [*n* (%)]	599 (64.41)	238 (59.80)	2.541	0.111
Statins [*n* (%)]	133 (14.30)	46 (11.56)	1.799	0.180
Antiplatelet [*n* (%)]	394 (42.37)	126 (31.66)	13.412	<0.001
Physical activity			4.376	0.112
Low intensity [*n* (%)]	352 (37.85%)	128 (32.16%)		
Medium intensity [*n* (%)]	123 (13.23%)	63 (15.83%)		
High intensity [*n* (%)]	455 (48.92%)	207 (52.01%)		
HR[times/minute, M(Q1,Q3)]	78.00 (71.00,86.00)	79.00 (72.00, 87.00)	1.851	0.064
Course [years, M(Q1,Q3)]	12.80 (7.60,17.60)	13.23 (9.79, 16.87)	1.433	0.152
LDL [mmol/L, M(Q1,Q3)]	3.26 (2.87,3.82)	3.34 (3.05, 3.64)	0.779	0.436
TG [mmol/L, M(Q1,Q3)]	1.70 (0.14,3.04)	1.26 (0.89, 1.77)	2.815	0.005
HDL [mmol/L, M(Q1,Q3)]	1.10 (0.82,1.42)	1.11 (0.89, 1.31)	0.432	0.666
TC [mmol/L, M(Q1,Q3)]	5.12 (3.67,6.84)	4.98 (4.29, 5.66)	1.660	0.097
BMI [kg/m^2^, M(Q1,Q3)]	21.81 (19.11,23.95)	22.05 (20.52, 23.42)	1.711	0.087
CRP [mg/L, M (Q1,Q3)]	13.00 (8.00,23.00)	10.00 (7.00, 14.00)	5.820	<0.001
PLT [×10^9^/L, M(Q1,Q3)]	264.00 (255.00,274.00)	251.00 (236.00, 265.00)	12.079	<0.001
UA [μmol/L, M (Q1,Q3)]	338.00 (309.00,373.00)	333.00 (315.00, 351.00)	1.905	0.058

M(Q1,Q3), median (first quartile, third quartile); *n*(%), number ((percentage); Non-CAS, non carotid atherosclerosis; CAS, carotid atherosclerosis; DM, history of diabetes mellitus; CIL, history of cerebral ischemic lesion; CHD, history of coronary heart disease; Drink, history of drinking; Smoke, history of smoking; Antihypertensive, use of antihypertensive drugs; Statins, use of statins; Antiplatelet, use of antiplatelet agents; Course, course of hypertension; PA, physical activity; HR, heart rate; LDL, low-density lipoprotein; TG, triglycerides; HDL, high-density lipoprotein; TC, total cholesterol; BMI, body mass index; CRP, C-reactive protein; PLT, platelet count; UA, uric acid.

### Comparison of clinical data between CAS group and non-CAS group in the development cohort

**Figure 1:**
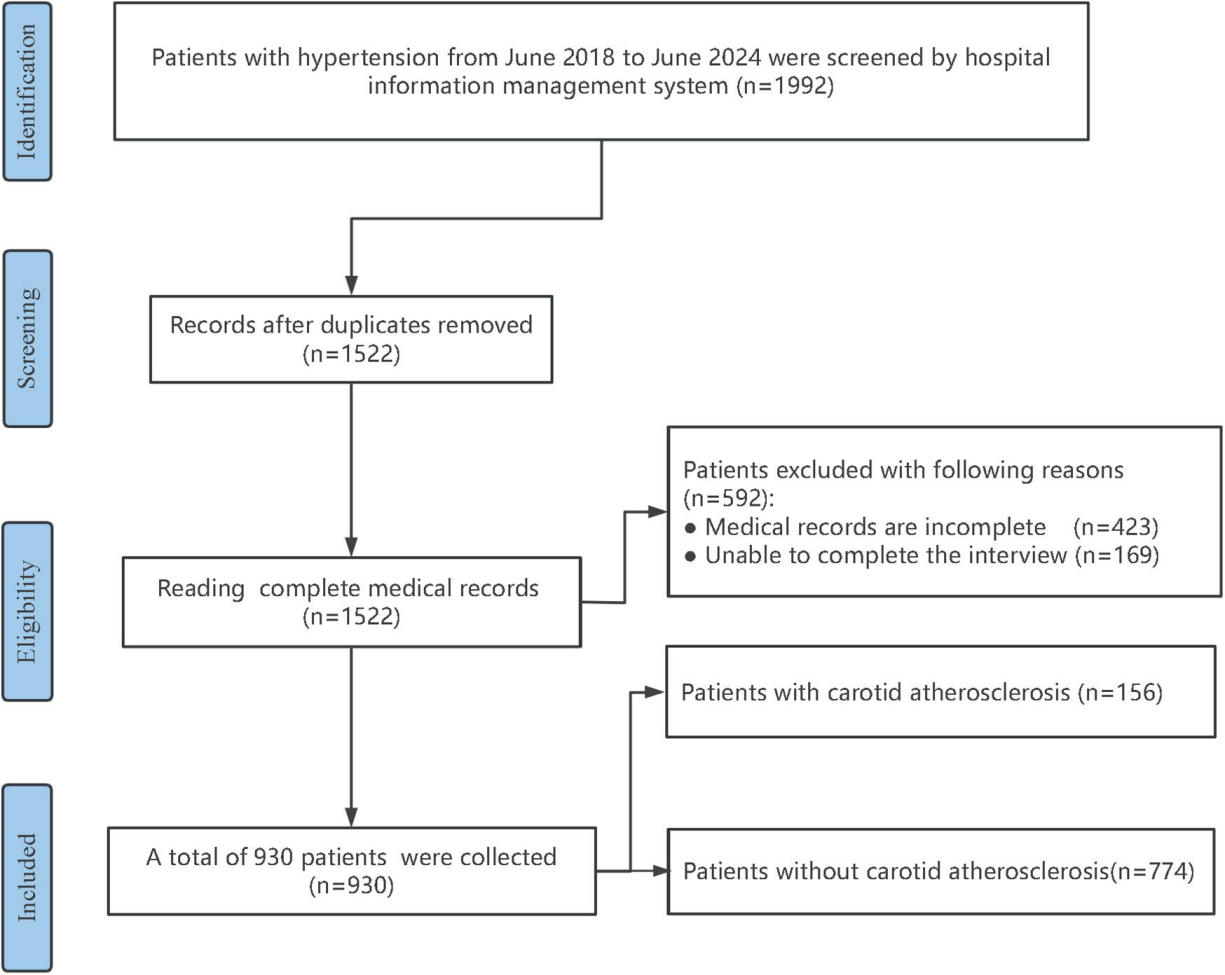
Flowchart of research subject screening.

A total of 930 hypertensive patients were enrolled in the development cohort and categorized into a CAS group (156 individuals) and a non-CAS group (774 individuals) based on the presence of CAS. The patient selection process is shown in Figure 1. The two groups were compared in terms of Age, male sex, DM, CHD, Smoke, Statins, PA, Course, Drink, LDL, BMI, CRP, and UA, etc., and the differences were statistically significant (*P* < 0.05). As shown in Table 2, compared with the non-CAS group, the CAS group was older and had a higher proportion of males, DM, CHD, Statin, Smoke, and Drink (all *P* < 0.05). The LDL, BMI, CRP, UA, and Course in the CAS group were higher than those in the non-CAS group (*P* < 0.05), while the proportion of high-intensity PA was lower than that in the non-CAS group (*P* < 0.05).

**Table 2:** Comparison of clinical data between CAS group and non-CAS group.

Variables	Non-CAS group (774 individuals)	CAS group (156 individuals)	Z/χ^2^	*P*-value
Age [years, M(Q1,Q3)]	55.00 (50.00, 62.00)	74.00 (71.00, 79.00)	17.868	<0.001
Male [*n* (%)]	414 (53.48)	82 (56.56)	5.730	0.017
DM [*n* (%)]	259 (33.46)	92 (58.97)	7.434	0.007
CIL [*n* (%)]	376 (48.57)	72 (46.15)	1.540	0.214
CHD [*n* (%)]	243 (31.39)	56 (35.89)	6.737	0.009
Drink [*n* (%)]	128 (16.53)	30 (19.23)	5.076	0.024
Smoke [*n* (%)]	157 (20.28)	45 (28.84)	10.827	<0.001
Antihypertensive [*n* (%)]	490 (63.31%)	109 (69.87%)	2.440	0.118
Statins [*n* (%)]	99 (12.79%)	34 (21.79%)	8.589	0.003
Antiplatelet [*n* (%)]	338 (43.67%)	56 (35.90%)	3.212	0.073
PA			37.917	<0.001
Low intensity [*n* (%)]	263 (33.98%)	89 (57.05%)		
Medium intensity [*n* (%)]	98 (12.66%)	25 (16.03%)		
High intensity [*n* (%)]	413 (53.36%)	42 (26.92%)		
HR [times/minute, M (Q1,Q3)]	78.00 (71.00, 86.00)	79.00 (69, 90)	0.545	0.586
Course [years, M(Q1,Q3)]	12.00 (7.00, 16.00)	24.00 (14.00, 36.00)	11.675	<0.001
LDL [mmol/L, M (Q1,Q3)]	3.20 (2.84, 3.68)	3.80 (3.14, 4.40)	7.330	<0.001
TG [mmol/L, M (Q1,Q3)]	1.70 (0.20, 3.01)	1.70 (0.00, 3.34)	0.449	0.653
HDL [mmol/L, M (Q1,Q3)]	1.10 (0.82, 1.42)	1.10 (0.81, 1.42)	0.017	0.986
TC [mmol/L, M(Q1,Q3)]	5.12 (3.69, 6.79)	5.12 (3.30, 6.88)	0.355	0.723
BMI [kg/m^2^, M (Q1,Q3)]	21.20 (18.54, 23.17)	24.80 (22.85, 26.83)	13.411	<0.001
CRP [mg/L, M (Q1,Q3)]	12.00 (7.00, 20.00)	21.00 (12.00, 34.75)	7.170	<0.001
PLT [×10^9^/L, M (Q1,Q3)]	263.00 (255.00, 273.00)	266.00 (257.00, 274.00)	1.063	0.288
UA [μmol/L, M(Q1,Q3)]	334.00 (309.00, 359.25)	488.00 (319.25, 613.75)	8.878	<0.001
		109 (69.87%)		

M(Q1,Q3), median (first quartile, third quartile); *n*(%), number ((percentage); Non-CAS, non carotid atherosclerosis; CAS, carotid atherosclerosis; DM, history of diabetes mellitus; CIL, history of cerebral ischemic lesion; CHD, history of coronary heart disease; Drink, history of drinking; Smoke, history of smoking; Antihypertensive, use of antihypertensive drugs; Statins, use of statins; Antiplatelet, use of antiplatelet agents; Course, course of hypertension; PA, physical activity; HR, Heart rate; LDL, low-density lipoprotein; TG, triglycerides; HDL, high-density lipoprotein; TC, total cholesterol; BMI, body mass index; CRP, C-reactive protein; PLT, platelet count; UA, uric acid.

### Results of LASSO regression and logistic regression

**Figure 2:**
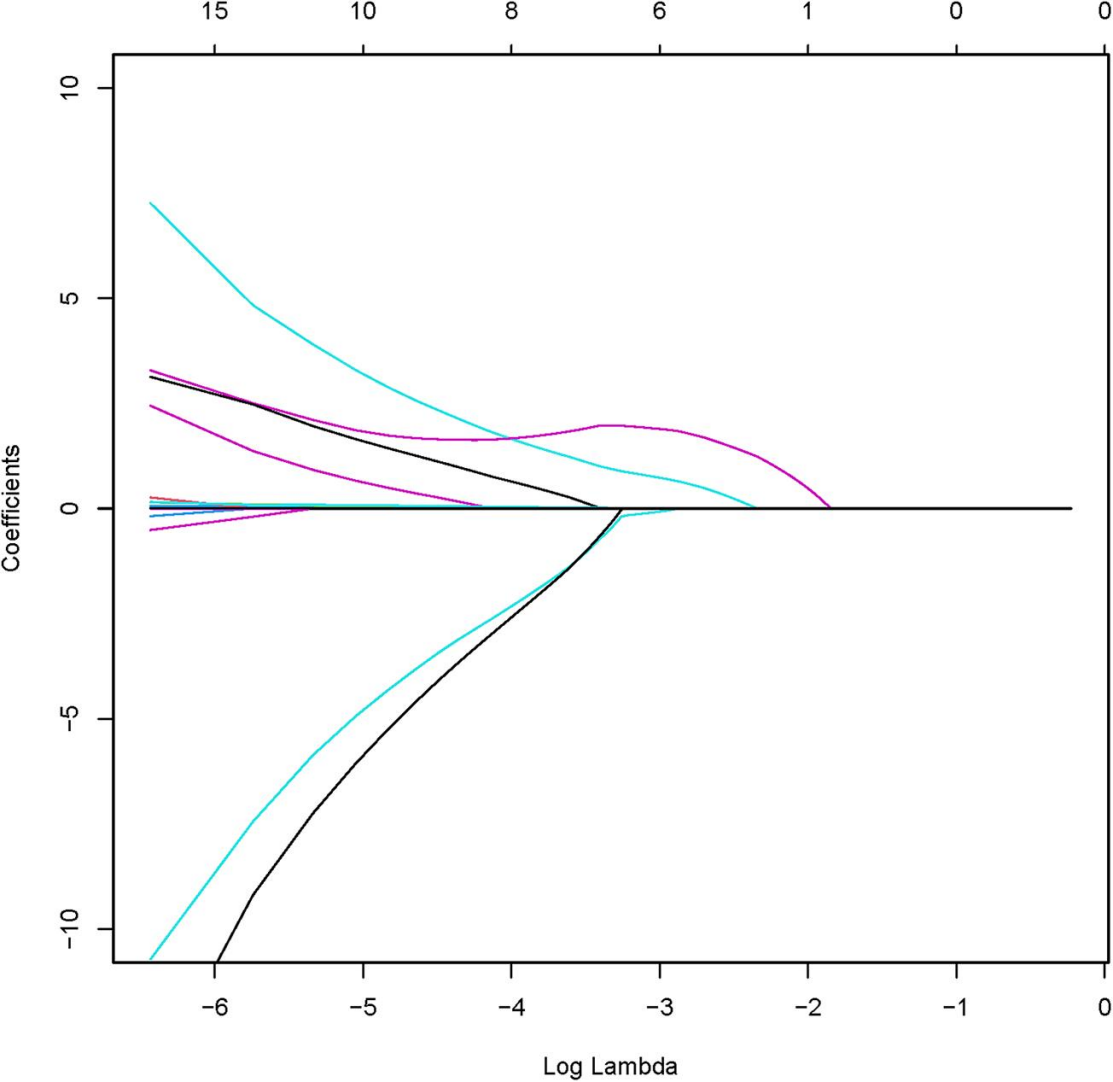
Coefficient path of LASSO regression.

**Figure 3:**
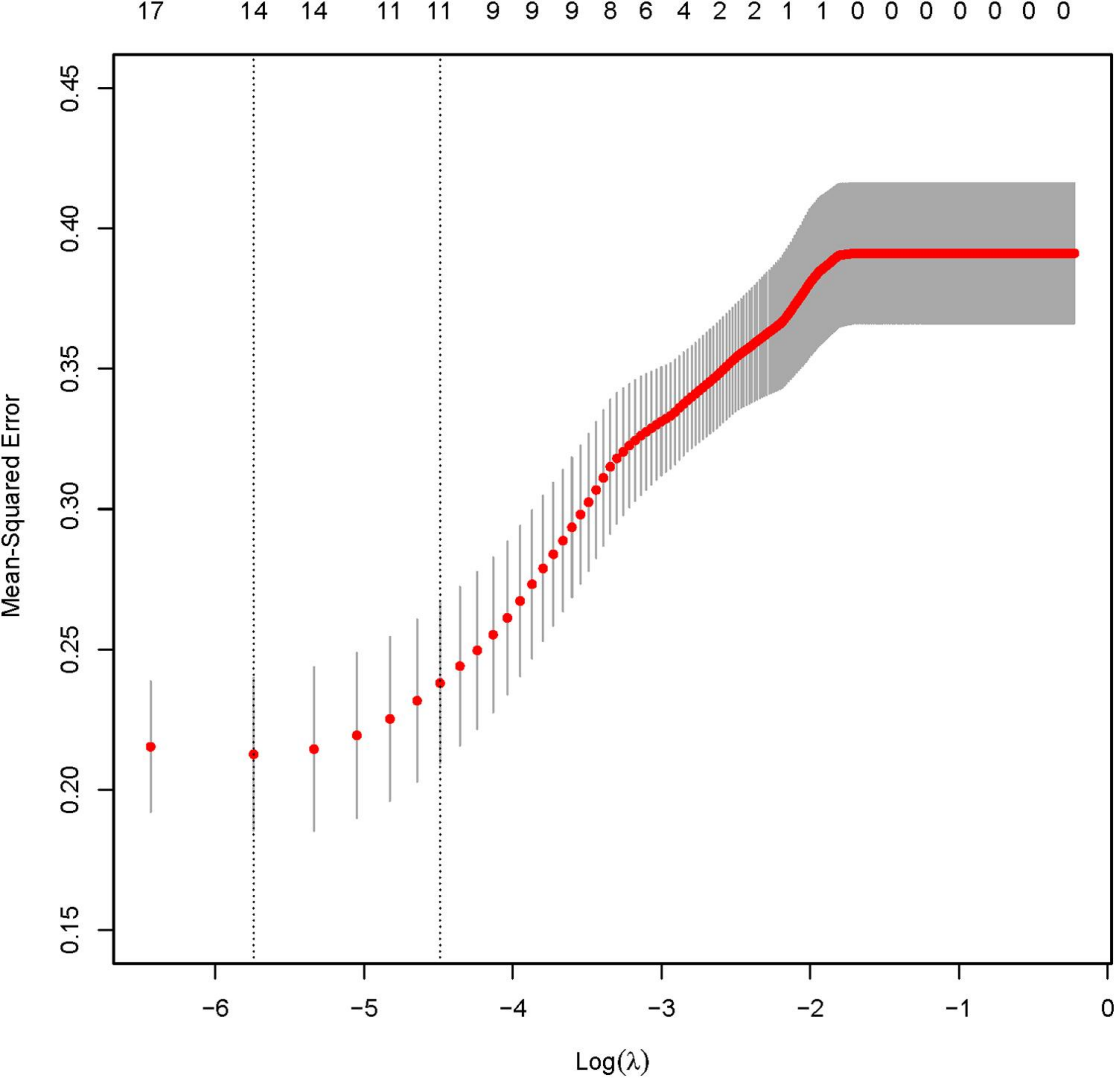
LASSO regression verification results.

**Figure 4:**
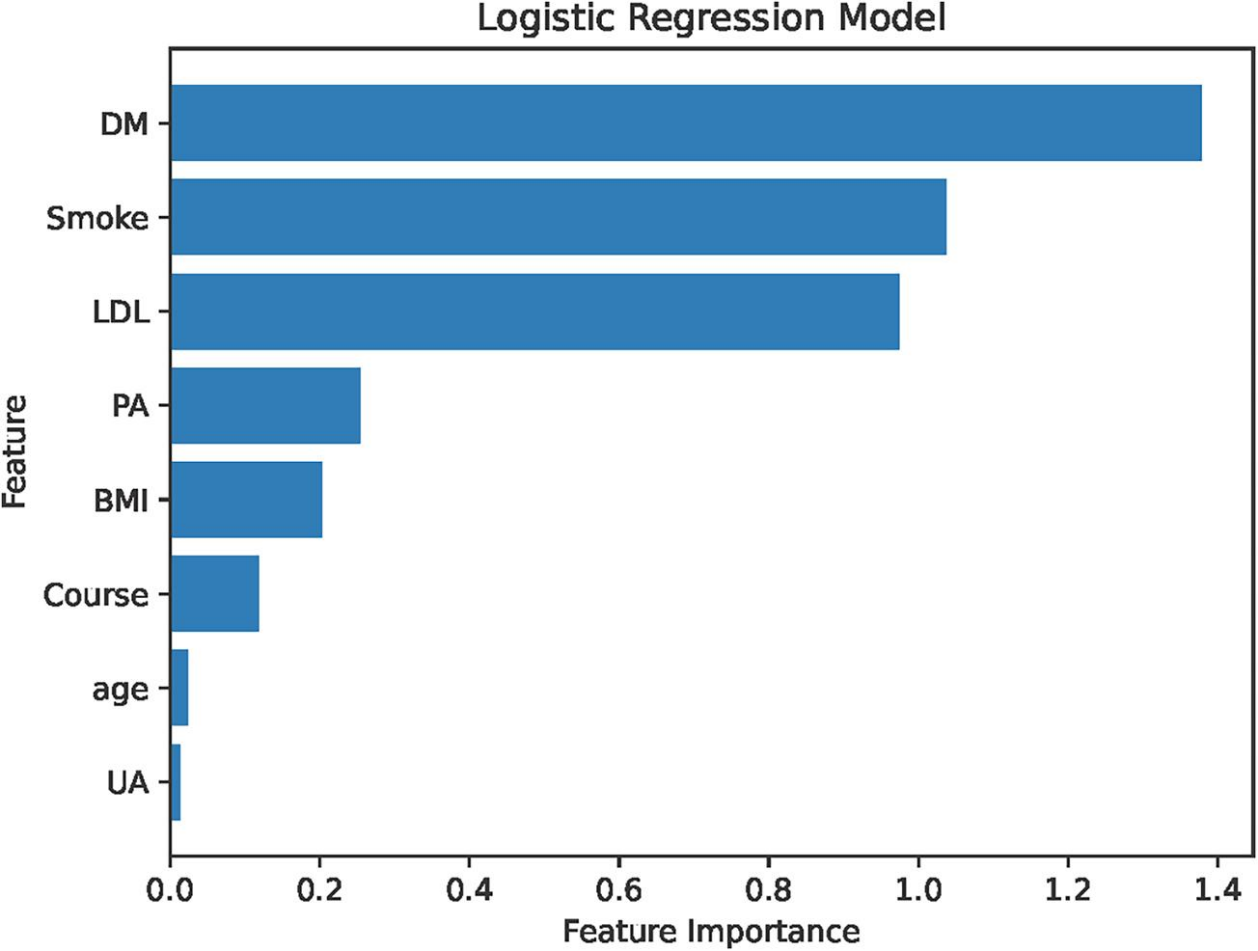
Importance ranking of risk factors. LDL, low-density lipoprotein; Course, course of hypertension; DM, history of diabetes mellitus; PA, physical activity; Smoke, history of smoking; UA, uric acid; CRP, C-reactive protein; BMI, body mass index.

The analysis designated the presence of CAS as the dependent variable, while the clinical variables that differed between the two patient groups served as independent variables. Initially, LASSO regression with 10-fold cross-validation was performed to optimize the regularization parameter (lambda). The cross validation curve shows that when lambda.1se = 0.15, the model reaches the optimal balance, at which the errors of both the training set and the validation set are the smallest. This process identified eleven candidate risk factors: Age, Sex, DM, Smoke, PA, Course, Drink, LDL, BMI, CRP, and UA (Figures 2, 3). Subsequent logistic regression further refined these factors, revealing that Age, DM, Smoke, PA, Course, LDL, BMI, and UA significantly contributed to the risk of CAS in hypertensive patients (see Table 3). Finally, using the feature_importances_function, the relative importance of these risk factors was computed and ranked as follows: DM, Smoke, LDL, PA, BMI, Course, Age, and UA I (as shown in Figure 4).

**Table 3:** Multivariate logistic regression analysis.

Variables	*β*	SE	Wald	OR (95% CI)	*P*-value
Age	0.099	0.031	10.465	1.104 (1.040–1.173)	0.001
LDL	0.320	0.150	4.969	1.377 (1.012–1.785	0.038
Course	0.080	0.028	8.369	1.083 (1.026–1.143)	0.004
PA					
Low	-	-	-	Reference	-
Medium	−1.200	0.550	4.76	0.301 (0.102–0.885)	0.031
High	−1.400	0.680	4.24	0.247 (0.065–0.937)	0.043
DM	1.200	0.420	8.162	3.320 (1.458–7.558)	0.004
Smoke	0.950	0.450	4.456	2.586 (1.070–6.249)	0.035
CHD	0.157	0.181	0.753	1.171 (0.820–1.671)	0.386
UA	0.005	0.002	7.059	1.005 (1.001–1.009)	0.008
CRP	0.188	0.634	0.087	1.207 (0.348–4.184)	0.767
BMI	0.281	0.109	6.589	1.324 (1.069–1.640)	0.010
Drink	0.035	0.037	0.900	1.036 (0.963–1.113)	0.343

β, regression coefficient; LDL, low-density lipoprotein; Course, course of hypertension; DM, history of diabetes mellitus; PA, physical activity; Drink, history of drinking; Smoke, history of smoking; CHD, history of coronary heart disease; UA, uric acid; CRP, C-reactive protein; BMI, body mass index; SE, standard error; OR, odds ratio; CI, confidence internal.

### Construction of the nomogram model

**Figure 5:**
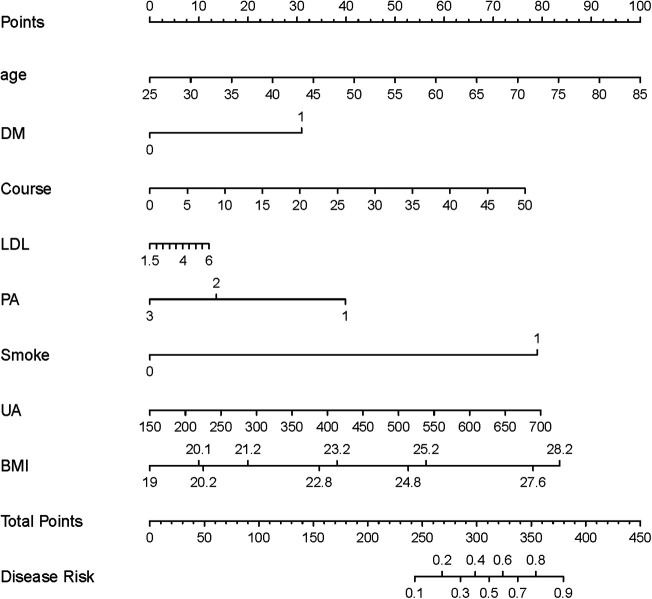
The nomogram model for predicting CAS risk in hypertensive patients. LDL, low-density lipoprotein; Course, course of hypertension; DM, history of diabetes mellitus; PA, physical activity; Smoke, history of smoking; UA, uric acid; CRP, C-reactive protein; BMI, body mass index.

Eight risk factors—Age, DM, PA, Smoke, Course, LDL, BMI, and UA—were identified to develop a nomogram for forecasting CAS risk in hypertensive patients (see Figure 5). Moreover, to enhance its clinical applicability, an interactive dynamic version of the nomogram is available at: https://cxf12345.shinyapps.io/DynNomapp/.

### Validation of the nomogram model

**Figure 6:**
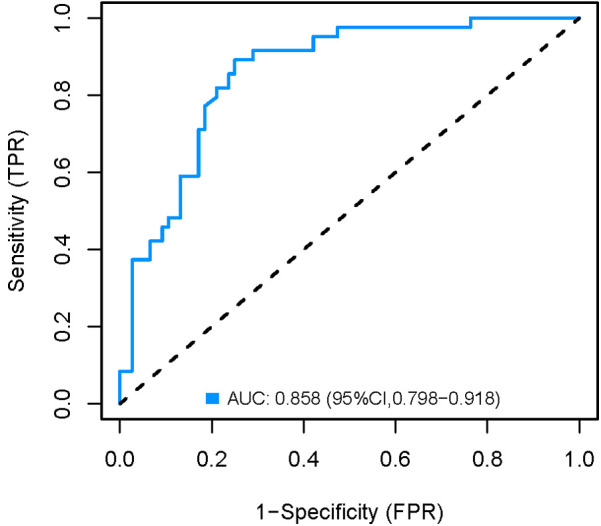
Internal validation AUC curve of the nomogram model.

**Figure 7:**
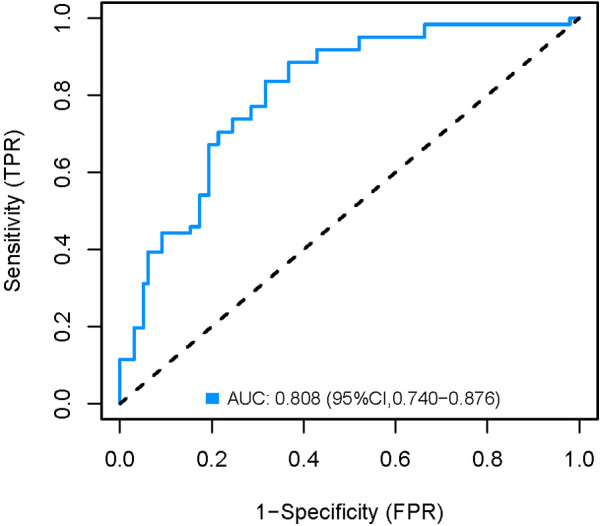
External validation AUC curve of the nomogram model.

The development cohort was internally validated using the Bootstrap resampling technique with 1,000 rereads. The results showed that the AUC of the model was 0.858 [95% CI (0.798, 0.918)] (Figure 6), with a specificity of 0.750, a sensitivity of 0.892 and an accuracy of 0.824. By collecting 398 patients from the Second Affiliated Hospital of Soochow University as an external validation cohort, the AUC of the external validation model was 0.808 [95% CI (0.740, 0.876)] (Figure 7), with a specificity of 0.684 a sensitivity of 0.836 and an accuracy of 0.742. The internal and external validation results showed that the prediction model of the development cohort had robust discriminative ability. The optimal risk threshold for clinical intervention was 0.30 (sensitivity: 82%, specificity: 75%), determined via Youden's index.

**Figure 8:**
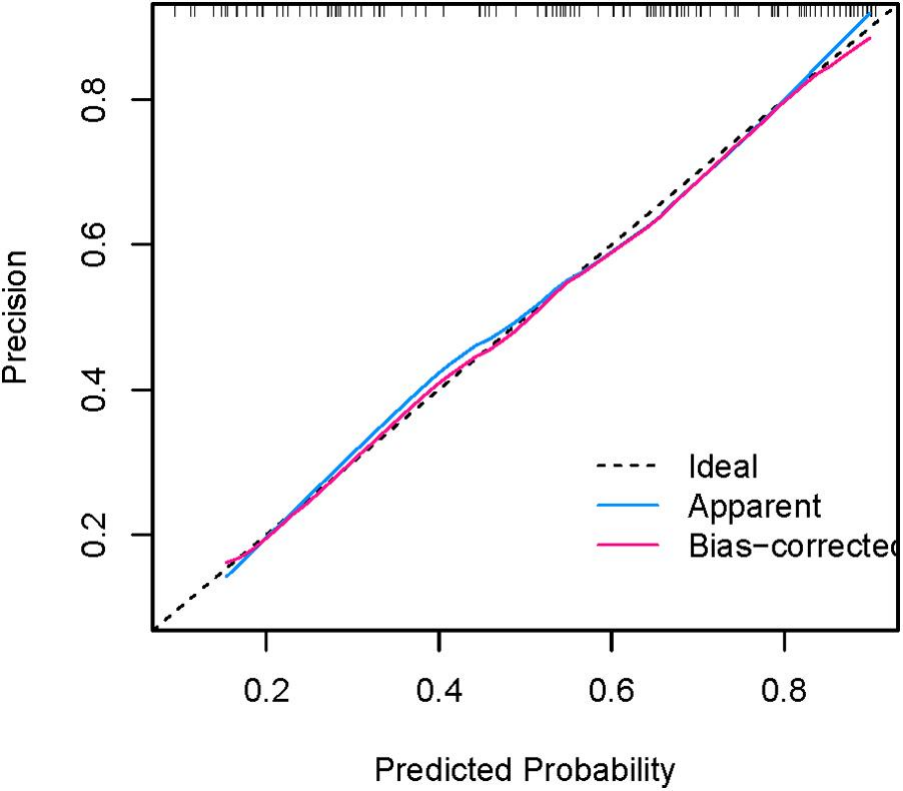
Calibration curve for internal validation of the nomogram model.

**Figure 9:**
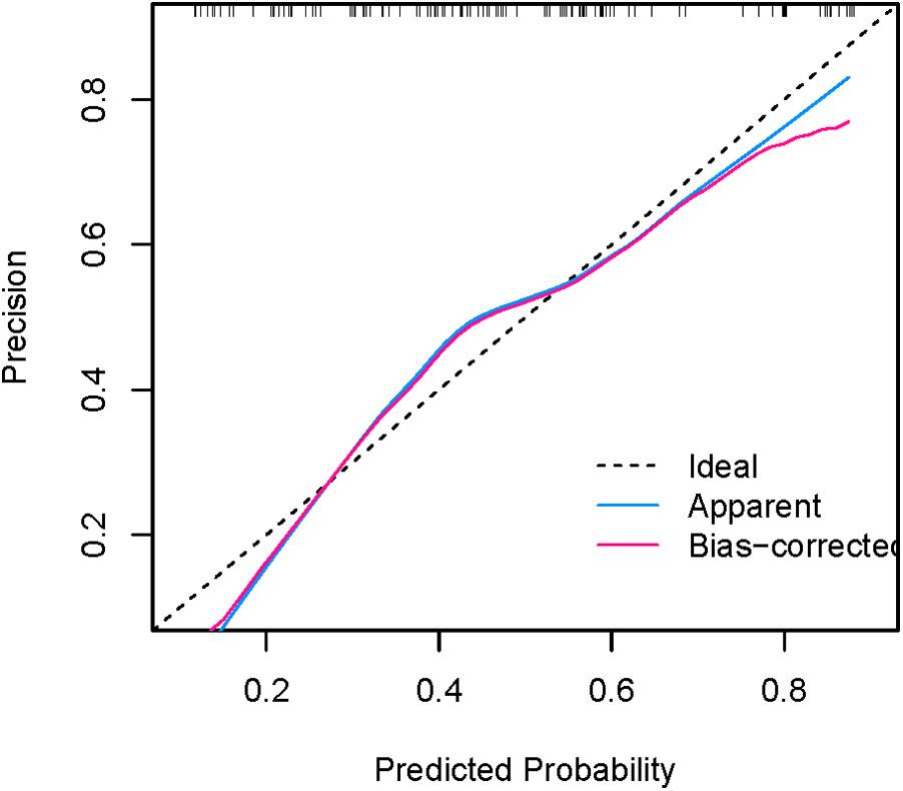
Calibration curve for external validation of the nomogram model.

**Figure 10:**
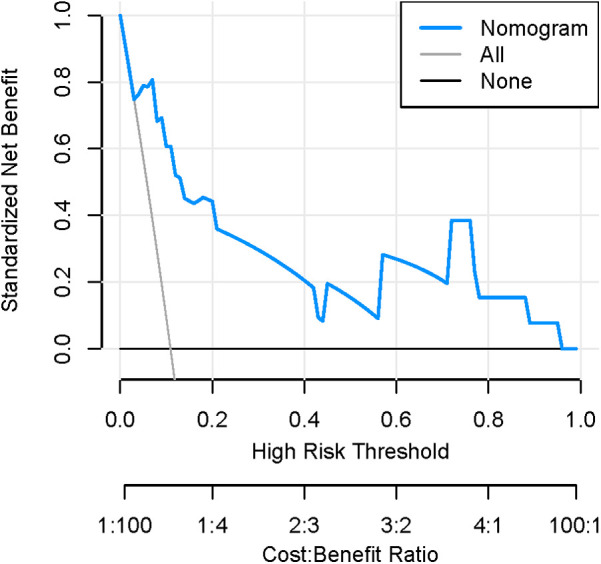
Decision curve of the nomogram model.

Figures 8, 9 show that the calibration curves of the development cohort and validation cohort coincided well with the ideal line, indicating that there was good consistency between the predicted probability and the observed probability. In addition, the Hosmer-Lemeshow goodness-of-fit test [*χ*^2^ = 9.864 (*P* = 0.274) for the development cohort model and *χ*^2^ = 6.177 (*P* = 0.624) for the validation cohort model] further confirmed the robustness of the model. Figure 10 indicates that the development cohort model performs well when the prediction probability threshold is 0.052–0.981.

### Subgroup analysis

According to the maximum blood pressure values monitored by hypertensive patients, the patients were divided into Group A (hypertension stage 1 group, BP max159/99 mmHg), Group B (hypertension stage 2 group, BP max 179/109 mmHg) and Group C (hypertension stage 3 group, BP max ≥180/110 mmHg). The grouping results showed that there were 342 individuals in Group A, 367 individuals in Group B and 221 individuals in Group C. Separate logistic regression models were developed for each group. Analysis revealed that DM, Smoke, LDL, PA, and BMI were consistent risk factors for CAS across all groups. In addition, Group A's model identified Age, Statins, and UA as risk factors, Group B's model incorporated Course, and Group C's model included Age, Drink, and Course. All models demonstrated excellent accuracy, with scores of 0.779 for Group A, 0.756 for Group B, and 0.802 for Group C. Interaction tests confirmed significant differences in risk factors across hypertension grades (P-interaction <0.05 for Age, Course, and Drink).

## Discussion

### Summary of findings

This study identified key risk factors for carotid atherosclerosis (CAS) in hypertensive patients and developed a predictive nomogram model with strong discriminative and calibration performance. Our findings highlight the multifactorial nature of CAS development in this high-risk population, emphasizing the roles of metabolic, inflammatory, and lifestyle-related factors. Below, we discuss the clinical implications of our results, compare them with previous literature, and outline the strengths and limitations of our study.

The variable selection method of this study adopts LASSO regression combined with 10-fold cross validation. This method can not only effectively deal with the multicollinearity problem in high-dimensional data, but also identify the most predictive variable combination through regularized path analysis (15). This approach identified eleven factors influencing CAS in hypertensive patients, which were further analyzed through multivariate logistic regression. The results confirmed that Age, DM, Smoke, Course, LDL, BMI, UA and PA are independent risk factors for CAS.

Studies indicate that CAS incidence rises with age, particularly after 40, accelerating past 49, underscoring a strong correlation between age and CAS (16). Our findings align with this, identifying advanced age as a key risk factor. DM contributes to CAS through glycated hemoglobin, which induces vascular endothelial dysfunction and oxidative stress. Prior research has established a positive link between glycated hemoglobin levels and CAS (17), supported by meta-analyses linking blood glucose levels to carotid intima-media thickness (18, 19). Our study corroborates these findings. Smoke remains a well-established CAS risk factor, contributing to systemic inflammation, endothelial impairment, and oxidative stress (20). Meta-analyses confirm its strong association with peripheral arterial sclerosis, particularly carotid atherosclerosis (21), with secondhand Smoke also posing a significant risk (22). Our study reaffirms Smoke as a major contributor to CAS in hypertensive patients. Hypertension itself fosters CAS through vascular endothelial damage and oxidative stress, with prolonged disease duration exacerbating the risk (23). Similarly, LDL plays a crucial role in CAS pathology, with lower levels linked to reduced plaque formation (24, 25). Meta-analyses highlight LDL as a key modifiable factor in carotid atherosclerosis (26), consistent with our findings. Despite lower TG levels in the validation cohort, the model maintained robust performance (AUC: 0.808), suggesting generalizability across populations with varying metabolic profiles (27). Obesity, a complex metabolic disorder, is another critical CAS determinant. It disrupts lipid metabolism, promotes insulin resistance, and triggers inflammation, all of which contribute to atherosclerosis (28). Research shows that weight loss interventions, particularly surgical procedures, can mitigate CAS progression (29, 30). Our study identifies elevated BMI as a significant CAS risk factor, reinforcing prior evidence. Hyperuricemia, through increased reactive oxygen species and altered intracellular signaling, promotes atherosclerotic lesions (31). Meta-analyses establish a strong correlation between serum uric acid levels and carotid intima-media thickness (32), with urolithiasis further elevating the risk of coronary and carotid atherosclerosis (33). Our findings confirm UA as an independent CAS risk factor in hypertensive patients. Our study found that low-intensity PA was significantly associated with CAS, corroborating evidence that sedentary behavior exacerbates arterial stiffness (34). Notably, high-intensity PA was protective, supporting current guidelines recommending moderate-to-vigorous exercise for cardiovascular risk reduction (35). These results highlight the need for tailored exercise interventions in hypertensive populations to prevent CAS progression. To mitigate CAS risk, effective strategies include hypertension management, glycemic control, smoking cessation, LDL and UA reduction, weight regulation, and tailored exercise interventions. These measures are essential in preventing CAS among hypertensive individuals.

Subgroup analysis revealed that while DM, smoking, LDL, PA, and BMI were consistent risk factors across all hypertension grades, predictive factors differed significantly based on hypertension severity. Specifically, Age, statin use, and UA emerged as important risk factors in grade 1 patients while Course played a key role in grade 2 patients. Notably, Drink (alcohol consumption) and Disease Course were identified as significant predictors for CAS occurrence specifically in grade 3 hypertension patients. These findings underscore the necessity of incorporating hypertension severity into CAS risk assessment and prevention strategies for hypertensive patients, advocating for personalized management approaches tailored to the specific grade of hypertension.

### Strengths and limitations

This study has several notable strengths. First, it evaluates CAS risk factors in hypertensive patients from multiple perspectives, including laboratory indicators, lifestyle factors, and medical history. Second, we developed an interactive online nomogram interactive nomogram (https://cxf12345.shinyapps.io/DynNomapp/) enables real-time risk stratification, surpassing static models in clinical utility. Third, the model incorporates previously overlooked factors such as UA, BMI and PA. Four, beyond traditional AUC analysis, we employed clinical decision and calibration curves to comprehensively assess the model's predictive performance, ensuring its practical applicability rather than focusing solely on accuracy (36). Lastly, The traditional scoring method for CAS, “Plaque-RADS score”, is mainly used to assess the risk of stroke in people who have already developed carotid plaques, while our nomogram is mainly used to assess the risk of carotid atherosclerosis in hypertensive people who do not have carotid plaques; The Plaque-RADS score is mainly based on the location, shape, size and other characteristics of the plaque under ultrasound imaging, while the scoring basis of the nomogram we developed is mainly Age, DM, Smoke, Course, LDL, BMI, UA and PA.

Despite these strengths, the study has certain limitations. As it exclusively examines hypertensive patients, the findings may not be generalizable to other populations, such as those with diabetes. While we considered twenty-one common risk factors, they do not encompass all potential influences on CAS, warranting further studies with a broader range of variables. The patients included in this study were all hospitalized patients, which may lead to an overestimation of the risk prediction value. In view of this, we plan to collect outpatients for prospective research in the future to further analyze the accuracy and practicality of the validation model. Additionally, the study's focus on Chinese patients from a single region limits its applicability to other demographics. Finally, the relatively small sample size and single-center design, though internally and externally validated, require confirmation through larger, multicenter prospective studies.

## Conclusions

This study established a nomogram model to predict the risk of CAS in hypertensive patients, which has certain clinical significance for the prevention and treatment of CAS in hypertensive patients.

## Data Availability

The raw data supporting the conclusions of this article will be made available by the authors, without undue reservation.
